# Transcranial direct current stimulation for learning and memory in Parkinson’s disease: a systematic review

**DOI:** 10.3389/fnins.2026.1931282

**Published:** 2026-09-08

**Authors:** Carlos Caudet-Pastor, Daniel López-Malo, Aina Luque-García

**Affiliations:** 1 Department of Psychology, Faculty of Health Sciences, Universidad Europea de Valencia, Valencia, Spain; 2 Faculty of Health Sciences, Universidad Europea de Valencia, Valencia, Spain

**Keywords:** learning, memory, Parkinson’s disease, tDCS, transcranial direct current stimulation

## Abstract

**Background:**

Cognitive impairment affecting memory and learning processes is a common non-motor feature of Parkinson’s disease (PD) and contributes substantially to functional decline and a reduced quality of life. Transcranial direct current stimulation (tDCS) has emerged as a potentially beneficial non-invasive neuromodulation technique capable of modulating cortical plasticity and enhancing cognitive and motor performance. However, its specific effects on memory- and learning-related outcomes in PD remain unclear.

**Objective:**

To systematically review and synthesize the available evidence regarding the effects of tDCS on memory and learning processes in individuals with Parkinson’s disease.

**Methods:**

A systematic review was conducted following PRISMA guidelines, with the protocol registered in PROSPERO (CRD42024559548). PubMed, Scopus, and Web of Science were searched between September and December 2025. Randomized controlled trials in individuals with PD evaluating the effects of tDCS on memory-, learning-, or cognition-related outcomes were included. Study selection, data extraction, and risk-of-bias assessment (ROB-2) were performed systematically.

**Results:**

Nineteen studies (2006–2024) with sample sizes of 9–42 participants were included. Most protocols used 1–2 mA stimulation targeting the dorsolateral prefrontal cortex (DLPFC) and primary motor cortex (M1). Improvements were observed in working memory, verbal fluency, executive function, and processing speed, particularly in multi-session protocols combined with cognitive or motor training. M1 stimulation was associated with enhanced motor learning consolidation, whereas single-session interventions showed limited effects. Limitations include small samples, methodological heterogeneity, and limited follow-up.

**Conclusion:**

Current evidence suggests potential effects of tDCS in memory and learning processes in PD, although evidence remains limited and heterogeneous, especially when delivered in repeated sessions and combined with training targeting frontostriatal dysfunction. However, variability in protocols and study quality limits generalizability. Larger, well-controlled trials with standardized designs are needed to establish robust clinical recommendations.

**Systematic review registration:**

https://www.crd.york.ac.uk/PROSPERO/view/CRD42024559548, identifier CRD42024559548.

## 1 Introduction

Parkinson’s disease (PD) is the second most common neurodegenerative disorder after Alzheimer’s disease and the most common movement disorder worldwide. Its prevalence has increased significantly in recent decades, especially in individuals over 50, with peak incidence between 55 and 65 years of age and a higher incidence in men (Pereira et al., 2024; Zhu et al., 2024). It is a chronic, progressive, and irreversible condition that imposes substantial health care and societal costs (Hermanowicz and Edwards, 2015).

Pathophysiologically, PD is characterized by the degeneration of dopaminergic neurons in the nigrostriatal pathway, leading to reduced striatal dopamine levels and disruption of the neural circuits involved in motor control, especially the indirect pathway of the basal ganglia (Obeso et al., 2008; Vázquez-Vélez and Zoghbi, 2021). It is also associated with the formation of Lewy bodies, resulting from the abnormal accumulation of alpha-synuclein in the brain (Dickson, 2018; Poewe et al., 2017; Simon et al., 2020).

Although bradykinesia, resting tremor, and rigidity are the cardinal motor features of PD, the disease presents with heterogeneous symptoms that include cognitive and affective symptoms.

Cognitive impairment in PD varies, and two main pathways have been proposed to explain it (Robbins and Cools, 2014). The first involves a dopamine-dependent fronto-striatal alteration, arising from disruption of the circuits connecting frontal cortical regions with the basal ganglia, that affects executive functions such as planning, working memory, and cognitive flexibility; alterations in the functional connectivity of these circuits may precede detectable structural changes (Bugalho, 2014; Janvin et al., 2003; Jellinger, 2023). The second involves more widespread deficits associated with later cognitive decline and dementia, including attentional and memory impairment. In the early stages, declarative memory deficits are less prominent; however, alterations in learning and implicit memory processes dependent on basal ganglia dopaminergic function, such as habit and motor learning, are frequently observed (Foerde and Shohamy, 2011).

According to the Braak staging model, the caudal-to-rostral spread of alpha-synuclein pathology—from the brainstem and olfactory structures toward limbic and neocortical regions—parallels disease progression, with cortical involvement in the later stages corresponding to the emergence of cognitive impairment (Braak et al., 2003). Mild cognitive impairment (MCI) is a common non-motor symptom in patients with PD; it is a transitional stage between normal cognitive function and dementia and is considered one of the strongest predictors of dementia (Yu and Wu, 2022), affecting patients’ quality of life and overall prognosis. Recent studies confirm poorer performance in domains such as attention, executive functions, working memory, and verbal fluency, suggesting a bidirectional association between cognitive impairment and motor symptoms (Hoogland et al., 2018; Longo et al., 2024). Mild cognitive impairment (MCI) is one of the common non-motor symptoms in patients with PD and it is a transitional stage between normal cognitive functioning and dementia and is considered one of the strongest predictors of dementia (Yu and Wu, 2022), affecting patients’ quality of life and overall prognosis.

Current treatment strategies for PD primarily target core motor symptoms through pharmacological management; however, they have limitations in addressing disease progression and non-motor symptoms, such as cognitive impairment, and may also increase adverse effects. As a result, intensive research is exploring advanced therapeutic interventions for PD, like cell-based therapies, gene therapies or neuromodulation (Serva et al., 2022; Kumar et al., 2024).

Neuroplasticity, defined as the brain’s ability to reorganize its structural and functional connections, is essential for learning and memory. Factors such as age, physical exercise, and cognitive stimulation influence its optimal functioning (Seng et al., 2022; Marzola et al., 2023). In PD, cortico-striatal synaptic plasticity is impaired, affecting both long-term potentiation (LTP) and long-term depression (LTD), which impacts learning and memory processes (Jin and Costa, 2015; Lovinger, 2010; Madadi Asl et al., 2022). This evidence supports the need for interventions that promote plasticity, such as non-invasive brain stimulation (Popescu et al., 2024).

Among these techniques, transcranial direct current stimulation (tDCS) has emerged as a potentially beneficial and generally well-tolerated neuromodulation technique that uses low-intensity electrical currents (1–2 mA) delivered through anodal and cathodal electrodes. Anodal stimulation typically induces a partial depolarization of neuronal membranes, increasing cortical excitability and facilitating neuronal firing. In contrast, cathodal stimulation generally produces relative membrane hyperpolarization, decreasing cortical excitability and reducing the likelihood of neuronal firing. These polarity-dependent changes in cortical excitability may modulate the activity of distributed neural networks and promote synaptic plasticity mechanisms resembling long-term potentiation (LPT) and long-term depression (LPD) (Nitsche et al., 2003; Nitsche et al., 2008). tDCS may facilitate plastic reorganization and improve cognitive domains such as working memory, verbal fluency, and processing speed (Goldthorpe et al., 2020; Narmashiri and Akbari, 2025; Sandrini et al., 2016). In addition, it can induce sustained effects after stimulation, with some studies reporting effects that persist for several weeks after stimulation (Huo et al., 2020). Its safety is widely documented, with mild adverse effects such as tingling or transient headache (Khedr et al., 2014; Siegert et al., 2021).

Evidence from psychiatric, neurodegenerative, and healthy populations suggests that tDCS may modulate cognitive performance, particularly when combined with cognitive training (Bagattini et al., 2023; Begemann et al., 2020; Brunelin et al., 2018; Cheng et al., 2020; Leach et al., 2019; Majdi et al., 2022; Meléndez et al., 2023; Nissim et al., 2019). In PD, tDCS has been investigated for both motor and cognitive symptoms, with potentially beneficial findings reported for working memory (Boggio et al., 2006), theory of mind (Adenzato et al., 2019), verbal fluency (Mishra and Thrasher, 2021; Pereira et al., 2013), executive functions (Doruk et al., 2014), and motor skill consolidation (Broeder et al., 2019; Broeder et al., 2023). However, studies focusing specifically on memory- and learning-related outcomes in PD remain limited, and their findings are not fully consistent.

Although tDCS has been proposed as a non-pharmacological adjunct for cognitive rehabilitation in Parkinson’s disease, prior reviews addressing this evidence base have reported mixed and domain-specific findings regarding learning and memory outcomes (Cammisuli et al., 2022; Suarez-García et al., 2020; Vandendoorent et al., 2023). Taken together, these findings support the need for a systematic review focused specifically on learning and memory outcomes in Parkinson’s disease, because the available literature suggests possible benefit in selected paradigms but does not yet establish a consistent effect across tasks, stimulation parameters, or follow-up periods.

The available evidence on the effects of tDCS in Parkinson’s disease is characterized by substantial heterogeneity in stimulation parameters, including intensity, session duration, stimulation frequency, cortical targets, and the use of concomitant cognitive or motor training. This variability limits direct comparisons across studies and hinders the identification of optimal protocols for enhancing learning- and memory-related outcomes. Consequently, a focused synthesis of the evidence is needed to clarify the potential role of tDCS in these domains and to identify methodological and clinical factors that may influence treatment response.

Therefore, the aim of this systematic review was to synthesize and critically evaluate the available evidence regarding the effects of transcranial direct current stimulation (tDCS) on learning- and memory-related processes in individuals with Parkinson’s disease. Specifically, we examined the influence of different stimulation protocols, cortical targets, and intervention characteristics on declarative memory, working memory, executive processes associated with learning, and procedural motor learning outcomes.

## 2 Materials and methods

### 2.1 Study design

Systematic review.

### 2.2 Search strategy

The review was conducted and reported in accordance with the PRISMA 2020 statement (Page et al., 2021). The review question was formulated using the PICOS framework: in individuals with Parkinson’s disease (Population), what are the effects of transcranial direct current stimulation (Intervention), compared with sham stimulation, no intervention, or other control conditions (Comparator), on memory and learning outcomes (Outcomes), as evaluated in interventional studies including randomized controlled trials, crossover trials, and other prospective intervention designs (Study design)?

Searches were conducted in PubMed, Scopus, and Web of Science databases between September 1, 2025, and December 31, 2025. No restrictions were applied regarding the publication date of the articles. The search was restricted to articles published in English or Spanish. No additional filters were applied in order to find as many results as possible. In addition, the reference lists of the included studies were hand-searched to identify potentially relevant articles not captured by the electronic search; however, no additional eligible studies were identified through this process.

The search strategy consisted of a series of key terms related to the study topic and predefined to answer the PICOS question. Two reviewers (CCP and DLM) independently screened records, assessed full text eligibility, and extracted data. Disagreements were resolved through discussion and consensus. Rayyan software^1^ was used for the storage, organization, and management of all references to ensure a comprehensive and systematic search of the available literature.

First, relevant free-text terms and their synonyms were identified and combined using the Boolean operators AND and OR. The search equations used for each of the consulted sources are shown in Table 1.

**TABLE 1:** Search strategy.

Database	Search strategy
PubMed	[(Parkinson (title/abstract) or “Parkinson* disease” (title/abstract) or Parkinsonism (title/abstract) or “Parkinsonian disorders” (title/abstract) or “Parkinson-dementia syndrome” (title/abstract)] and [cognition (title/abstract) or cognitive (title/abstract) Or “cognitive impairment” (title/abstract) or memory (title/abstract) or learning (title/abstract) or “cognitive decline” (title/abstract) or “mental deterioration” (title/abstract)] and [“transcranial direct current stimulation” (title/abstract) or tDCS (title/abstract)]
Scopus	TITLE-ABS-KEY (Parkinson or “Parkinson* disease” or Parkinsonism or “Parkinsonian disorders” or “Parkinson-dementia syndrome”) and (TITLE-ABS-KEY (cognition or cognitive or “cognitive impairment” or memory or learning or “cognitive decline” or “mental deterioration”)] and (TITLE-ABS-KEY (“transcranial direct current stimulation” or tDCS)]
Web of science	[TS = (Parkinson or “Parkinson* disease” or Parkinsonism or “Parkinsonian disorders” or “Parkinson-dementia syndrome”) and TS = (cognition or cognitive or “cognitive impairment” or memory or learning or “Cognitive Decline” or “Mental Deterioration”) and TS = (“transcranial direct current stimulation” or tDCS)]

### 2.3 Study selection

We included studies that applied tDCS to individuals diagnosed with Parkinson’s disease and that included neuropsychological measures of learning and memory. No restrictions were applied regarding age, comorbid conditions, gender, or ethnicity. The inclusion criteria were:

1. Individuals diagnosed with Parkinson’s disease.
2. Studies employing transcranial direct current stimulation (tDCS) applied to the cerebral cortex.
3. Interventional studies evaluating tDCS in individuals with Parkinson’s disease, including randomized controlled trials, crossover trials, and non-randomized prospective studies.
4. Use of at least one neuropsychological measure related to memory and/or learning. Given the strong dependence of memory and learning processes on executive and attentional systems in Parkinson’s disease, studies assessing working memory, executive functions, verbal fluency, and procedural learning were also considered eligible.
5. Articles published in English or Spanish.

On the other hand, the following restrictions were applied regarding exclusion criteria:

1. Studies including participants with dementia unrelated to Parkinson’s disease, unless data for participants with PD were reported separately.
2. Use of other types of stimulation such as TMS or tACS, or the combination of these with tDCS.
3. Case studies, reviews, meta-analyses, clinical practice guidelines, protocols, or gray literature.
4. Articles without access to the full text.
5. Studies that did not include at least one neuropsychological outcome related to memory and/or learning.

### 2.4 Screening and data extraction

First, a search was conducted in the aforementioned databases, and the results were imported into Rayyan software to identify and exclude any potential duplicates from the search process. After duplicate removal, titles and abstracts were screened against the eligibility criteria. Full texts of potentially relevant articles were then assessed for inclusion. Two reviewers independently performed study selection and data extraction, and disagreements were resolved through discussion and consensus. A standardized data extraction form was used to collect information on study characteristics, sample size, participant characteristics, neuropsychological outcomes related to memory and learning, tDCS protocol parameters, and main findings.

Risk of bias was assessed independently by two reviewers (CCP and DLM) using the Cochrane Risk of Bias 2 (RoB 2) tool. Disagreements in domain ratings or overall judgments were resolved through discussion and consensus. The assessment considered the five ROB-2 domains: randomization process, deviations from intended interventions, missing outcome data, measurement of the outcome, and selection of the reported result. Overall judgments were assigned according to the recommended ROB-2 algorithms. To visualize the results, traffic-light and summary plots were generated.

## 3 Results

### 3.1 Study selection

**FIGURE 1:**
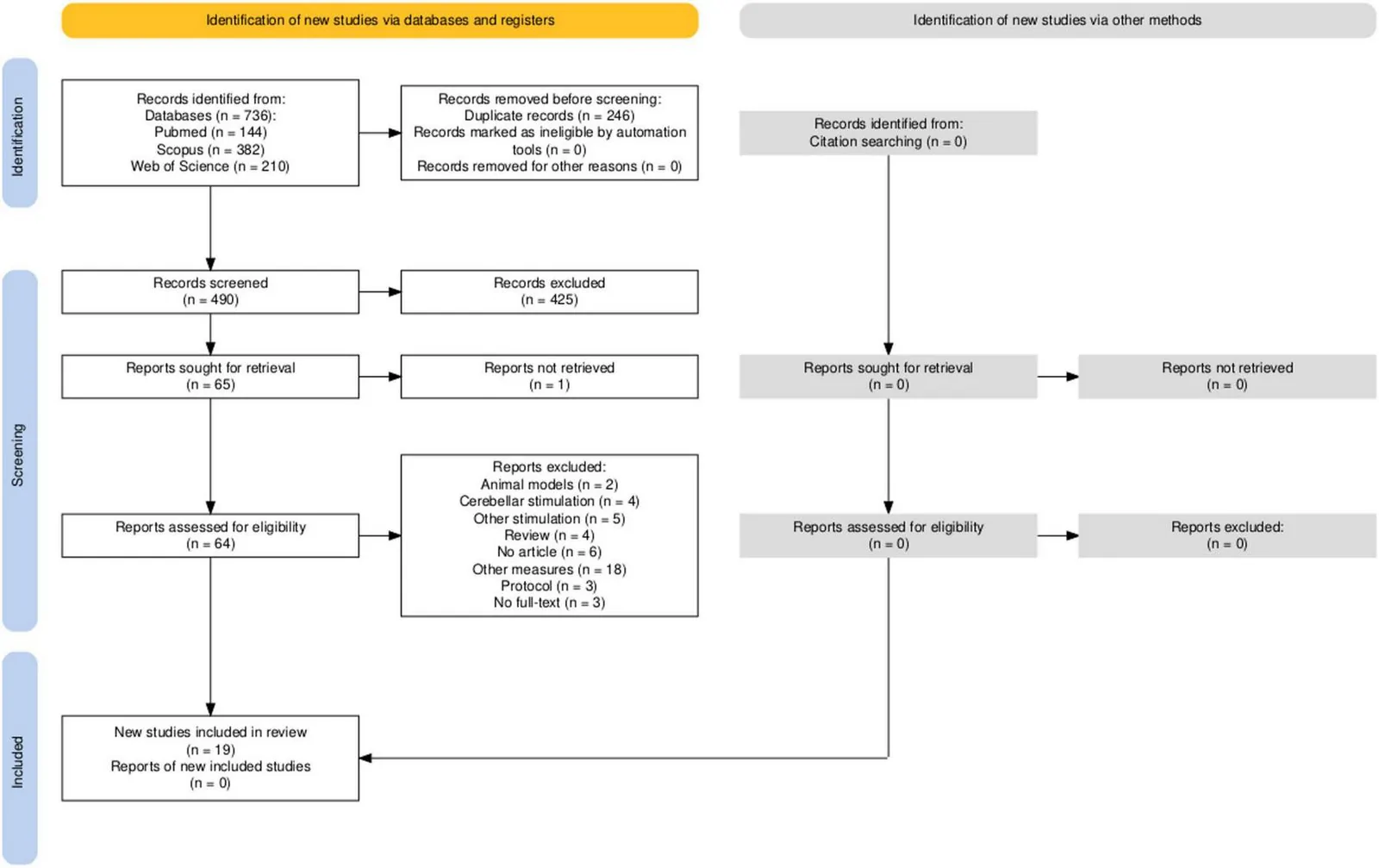
PRISMA 2020 flow diagram of the study selection process.

The study selection process is presented in Figure 1 using the PRISMA 2020 flow diagram.

### 3.2 Study characteristics

This systematic review included 19 studies that evaluated the effects of transcranial direct current stimulation (tDCS) on memory- and learning-related outcomes in individuals with Parkinson’s disease. The studies were conducted between 2006 and 2024, with sample sizes ranging from 9 to 42 participants. The study designs were primarily randomized, sham-controlled clinical trials, some with a crossover design (e.g., Broeder et al., 2019; Firouzi et al., 2021; Lau et al., 2019).

### 3.3 tDCS protocol characteristics

The stimulation intensity ranged from 1 mA to 2 mA, with 2 mA being the most common in the reviewed protocols. For example, Boggio et al. (2006) applied 2 mA for 20 min to the left DLPFC, which produced significant improvements in working memory. Similarly, Manenti et al. (2018) used 2 mA for 25 min over 10 sessions, combined with cognitive training, observing improvements in verbal fluency and declarative memory. The number of sessions ranged from a single application (Lau et al., 2019; Simpson and Mak, 2022) to extended protocols of 10–12 sessions (Dobbs et al., 2018; Manenti et al., 2018; Wong et al., 2024). The most frequently stimulated areas were the dorsolateral prefrontal cortex (DLPFC), the primary motor cortex (M1), and, to a lesser extent, the frontopolar area (FPA). The cathode was typically placed in the contralateral supraorbital region, except in the study by Ishikuro et al. (2018), where it was placed in the occipital region.

### 3.4 Memory and learning outcomes

Memory and learning outcomes were assessed using a range of standardized neuropsychological measures, such as the Trail Making Test (TMT-A and TMT-B), semantic and phonemic verbal fluency tests, the Rey Verbal Learning Test (RAVLT), the Letter-Number Sequencing (LNS) and the forward and backward digit tests from the Wechsler Memory Scale, as well as specific motor learning tasks and experimental paradigms of implicit memory. The TMT was used to assess processing speed and cognitive flexibility, with consistent improvements observed in Part B, associated with executive functions, in studies such as Doruk et al. (2014) and Bueno et al. (2019). Verbal fluency, both semantic and phonemic, was assessed in studies such as Manenti et al. (2018) and Mishra and Thrasher (2021) 2022, where the combination of tDCS and dual-tasking produced significant increases in the number of words generated per minute, reporting benefits in working memory and semantic declarative memory. Similar findings were reported by Lawrence et al. (2018), with participants in the active tDCS group exhibiting improved performance on both working memory and attentional tasks compared to the control condition. Similarly, Manenti et al. (2016) reported significant improvements in global cognition (PD-CRS), semantic verbal fluency, and executive functioning (TMT-B) following anodal DLPFC stimulation combined with physical therapy, with cognitive benefits maintained at the 3-month follow-up. Other tests, such as the RAVLT and the forward and backward digit sequences, were administered in the studies by Dobbs et al. (2018) and Manenti et al. (2018), with positive effects on immediate and delayed recall, although changes in verbal learning were not always detailed.

In the motor domain, tasks such as the Serial Reaction Time Task (SRT) were used to assess implicit sequential learning; Firouzi et al. (2021) reported significant improvements in consolidation 1 week after the intervention, on the consolidation of procedural memory. Manual dexterity tests such as the STEF were also used, in which Ishikuro et al. (2018) found that anodal stimulation over the frontopolar area improved performance on complex, high-dexterity tasks. Broeder et al. (2019) 2023 reported that the combination of tDCS over M1 and writing training increased stroke amplitude and speed, with sustained effects on retention and transfer to untrained tasks. On the other hand, Simpson and Mak (2022) evaluated explicit motor learning using finger-tapping tasks, observing no improvements with anodal tDCS over M1, though improvements were seen with placebo and cathodal conditions. Finally, some protocols that integrated tDCS with physical exercise or gait training, such as those by Conceição et al. (2021) and Wong et al. (2024), showed improvements in processing speed, coordination, and performance on cognitive dual-tasking.

The included studies were characterized by small sample sizes, heterogeneous inclusion criteria, variable stimulation parameters, and short follow-up periods.

Overall, the results indicate that the most robust improvements were obtained in protocols with multiple sessions and in those that combined tDCS with cognitive or motor training, particularly over the DLPFC for executive functions, declarative memory, and working memory, and over M1 for motor learning consolidation. In contrast, single-session studies, such as those by Lau et al. (2019) and Swank et al. (2016), showed no significant effects on memory or inhibitory control.

These findings should be interpreted cautiously given that most studies presented some concerns, particularly due to lack of preregistration and incomplete reporting of randomization procedures.

The detailed characteristics of each study, including stimulation parameters, design, and specific results, are presented in Table 2.

**TABLE 2:** Studies on memory and learning outcomes after tDCS in patients with PD (*n* = 19).

Reference	Patient sample size	tDCS		Study design	Memory and learning outcome measure	Key findings in learning and memory
		Intervention	Target			
Aksu et al. (2022)	26	Twice daily for 5 d, 20 min, 2 mA	L DLPFC	RCT double-blind between-subjects tDCS *n* = 13 Sham *n* = 13	WMS-IR/DR; COWAT; DS (F/B), VMPT; BNT	tDCS WMS-IR: + (post), + (1 m) tDCS WMS-DR: + (post), + (1 m) tDCS BNT: + (post), + (1 m)
Boggio et al. (2006)	18	Exp 1: single, 1 mA, 20 min. Exp 2: single, 2 mA, 20 min	L DLPFC or M1	Crossover RCT Exp 1: *n* = 9 Exp 2: *n* = 9	Three-back task (working memory)	L DLPFC 2 mA: +
Broeder et al. (2019)	10	Single tDCS, 20 min, 1 mA and single sham session after 3 w with writing training	L M1	Crossover RCT	Writing performance	Writing performance: + (post), + (1 w)
Broeder et al. (2023)	39	2 sessions, 20 min, 1 mA	L M1	Crossover RCT double-blind, tDCS *n* = 20 Sham *n* = 19	Writing performance with cognitive interference	Writing performance tDCS, medication “ON”: + (post), + (1 d)
Bueno et al. (2019)	20	Single tDCS session, 20 min, 2 mA and single sham session in 2 w	L DLPFC	RCT double-blind,	VFT; TMT-A/B	TMT-B: + VFT: +
Conceição et al. (2021)	20	Single tDCS session, 20 min, 2 mA with aerobic exercise and single sham session with aerobic exercise in 2 w	More affected DLPFC	Crossover RCT	MoCA; TMT-A/B; RT	Active tDCS and exercise: + (MoCA; TMT-A/B; RT) Sham tDCS and exercise: ± (RT); + (MoCA; TMT-A/B)
Doruk et al. (2014)	18	10 sessions for 2 w, 20 min, 2 mA	L or R DLPFC	RCT double-blind, between subjects L tDCS *n* = 6 R tDCS *n* = 5 Sham *n* = 7	TMT-A/B; DS (F/B); HVOT; n-back task	TMT-B: + (1 m)
Horiba et al. (2019)	18	2 sessions (1-week interval), 20 min, 2 mA	R M1	RCT double-blind sham-controlled Sham *n* = 9 Active tDCS *n* = 9	MMSE; frontal assessment battery; motor learning tasks	Motor learning: + (post), + (1 w)
Firouzi et al. (2021)	11	1 active tDCS session, 20 min, 2 mA and 1 sham	M1 dominant hand	Single-blind, sham-controlled, crossover	Serial reaction time (SRT)	SRT: + (1 w)
Ishikuro et al. (2018)	9	5 sessions per condition for 3 w, 15 min, 1 mA with physiotherapy	FPA	Crossover RCT (anodal, cathodal, and sham)	TMT-A; STEF	TMT-A: +Motor learning: +
Lau et al. (2019)	10	Single tDCS session, 20 min, 2 mA	L DLPFC	RCT between-subjects tDCS *n* = 5 Sham *n* = 5	Visual working memory and go/no-go test	Visual working memory and go/no-go test: ±
Lawrence et al. (2018)	42	1 session per week, for 4 w, 20 min, 1.5 mA	L DLPFC	RCT sham-controlled with 6 conditions (standard cognitive training, tailored cognitive training, tDCS + standard, tDCS + tailored, tDCS alone and control) *n* = 7 per condition	SOC; COWAT; LNS; HVLT-R; Paragraph Recall Test; BNT; MMSE	SOC: + COWAT: ± LNS: + HVLT-R: ± Paragraph Recall Test: + BNT: ± MMSE: ±
Manenti et al. (2016)	20	10 sessions (5 days/week for 2 weeks), 25 min, 2 mA, combined with physical therapy	DLPFC contralateral to the most affected side	RCT, double-blind, between-subjects. Anodal + PT *n* = 10 Sham + PT *n* = 10	PD-CRS; semantic fluency; TMT-B; digit span; PAL	PD-CRS total: + (post), + (3 m); PD-CRS frontal-subcortical: + (post), + (3 m); Semantic fluency: + (post), + (3 m); TMT-B: + (post), ± (3 m); Digit span: ± ; PAL: ±
Manenti et al. (2018)	22	5 sessions per week, over 2 week, 25 min, 2 mA	L DLPFC	RCT between subjects, double-blind. tDCS + CT *n* = 11 Sham + CT *n* = 11	RAVLT IR and DR; DS (F/B), VFT phonemic and semantic; TMT-A/B	VFT semantic: + (post), + (3 m) VFT phonemic: + (post)
Mishra and Thrasher (2022)	20	Single, 30 min, 2 mA	L DLPFC	RCT double-blind, crossover and sham-controlled	VFT; VFT—walking	tDCS VFT–walking: + (during), + (after), + (15 min)
Mishra and Thrasher (2021)	20	Single, 30 min, 2 mA	L DLPFC	RCT double-blind, crossover and sham-controlled	VFT; VFT—walking	VFT: + (15 min) VFT–walking: + (15 min), + (30 min).
Simpson and Mak (2022)	33	Single, 20 min, 2 mA	L M1	RCT single-blind, sham-controlled Anodal *n* = 11 Cathodal *n* = 11 Sham *n* = 11	Explicit motor sequence learning; skill index	Motor sequence learning (explicit): + (practice effect) Anodal tDCS: - Cathodal tDCS: + Sham: +
Swank et al. (2016)	10	Single tDCS, 20 min, 2 mA and single sham, 20 min over 2 weeks.	L DLPFC	Crossover RCT, single-blind	TUG and working memory task	tDCS interference: + tDCS or sham TUG and working memory task: ±
Wong et al. (2024)	34	12 Sessions (2–3 per week), 20 min, 2 mA	Dominant DLPFC	tDCS *n* = 17 Sham *n* = 17	Double task: working memory + walking	Active tDCS: + Sham tDCS: ±

BNT, Boston Naming Test; COWAT, Controlled Oral Word Association Test; CT, Cognitive Training, d day; DLPFC, Dorsolateral Prefrontal Cortex; DS (F/B), Digit Span (forward/backward); FPA, Frontopolar Area; HVLT-R, Hopkins Verbal Learning Test-Revised; HVOT, Hooper Visual Organization Test; L, Left; LNS, Letter-Number Sequencing; M1, Primary Motor Cortex; MMSE, Mini-Mental State Examination; MoCA, Montreal Cognitive Assessment; PD, Parkinson’s disease; R, Right; RAVLT, Rey Auditory Verbal Learning Test; RCT, Randomized Clinical Trial; RT, Reaction Time; SOC, Stockings of Cambridge; STEF, Standardized Test for Executive Functioning; TMT-A/B, Trail Making Test Parts A and B; TUG, Timed Up and Go; VFT, Verbal Fluency Task; VMPT, Virtual Multiple-Problem Task; w, week; WMS-IR, Wechsler Memory Scale Immediate Recall; WMS-DR, Wechsler Memory Scale Delayed Recall. The key findings were expressed by improvement “+,” no statistically significant change “±,” and negative impact “-”.

### 3.5 Risk of bias

**FIGURE 2:**
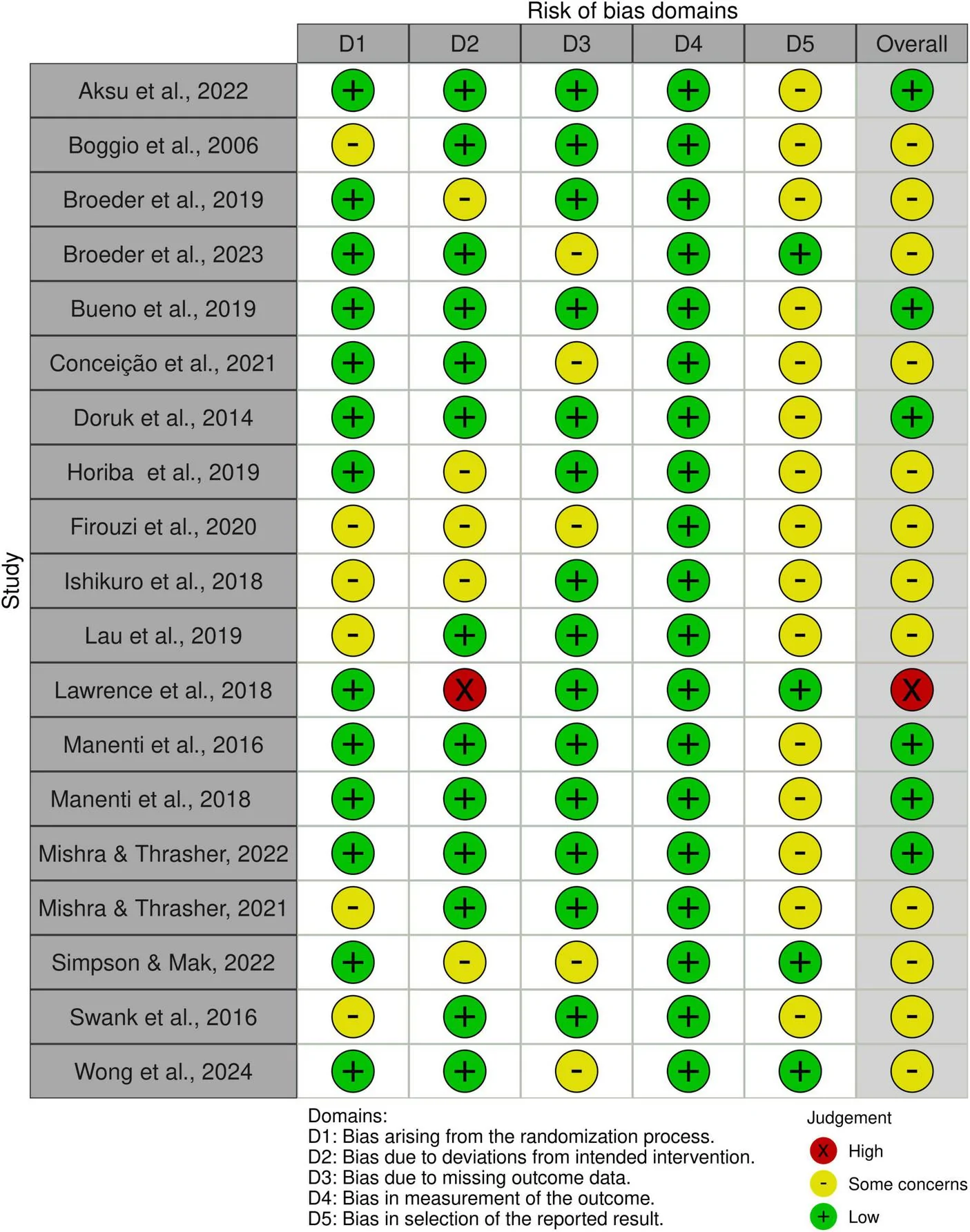
Risk of bias in randomized trials assessed with RoB-2. Traffic plot generated using robvis (McGuinness and Higgins, 2021).

**FIGURE 3:**
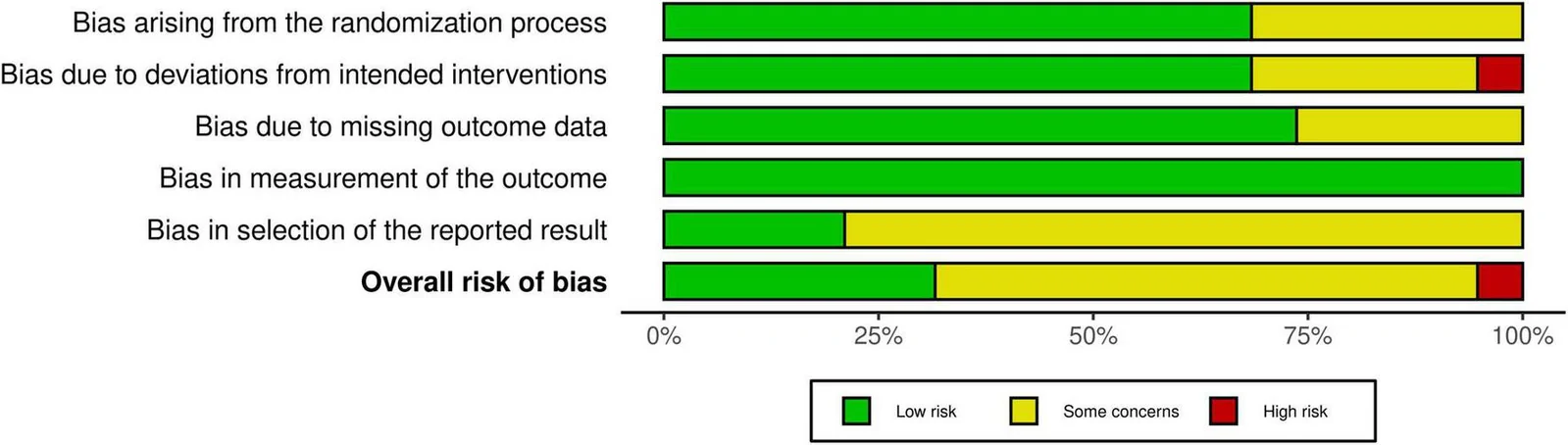
Risk of bias in randomized trials assessed with RoB-2. Summary plot generated using robvis (McGuinness and Higgins, 2021).

The methodological quality of the included studies was assessed using the Cochrane Risk of Bias 2 (ROB-2) tool, evaluating five domains: randomization process, deviations from intended interventions, missing outcome data, measurement of the outcome, and selection of the reported result. The ROB-2 results are summarized in Figures 2, 3.

Overall, most of the studies were classified as presenting “some concerns,” with a smaller proportion rated as low risk of bias and only one study considered at high risk of bias. This distribution reflects a body of literature with generally acceptable methodological rigor but important recurring limitations.

Regarding the randomization process (Domain 1), several recent randomized controlled trials adequately described sequence generation and allocation concealment procedures, such as the use of sealed envelopes or computerized randomization (e.g., Manenti et al., 2018; Mishra and Thrasher, 2022; Wong et al., 2024). However, several studies, particularly those using crossover designs, failed to provide sufficient details about sequence generation or concealment (e.g., Swank et al., 2016; Lau et al., 2019), leading to a classification of “some concerns” in this domain.

In the domain of deviations from intended interventions (Domain 2), most studies were rated as low risk, as they implemented appropriate double-blind procedures with well-established sham stimulation protocols (e.g., Aksu et al., 2022; Mishra and Thrasher, 2022). Nevertheless, some trials adopted single-blind designs or did not clearly report blinding of outcome assessors (e.g., Firouzi et al., 2021; Simpson and Mak, 2022), introducing potential performance or detection bias.

For missing outcome data (Domain 3), most studies reported low attrition rates and were therefore rated as low risk. However, some trials did not clearly describe the handling of missing data or lacking explicit intention-to-treat analyses, particularly in cases with participant exclusions or dropouts (e.g., Simpson and Mak, 2022; Wong et al., 2024). These studies were consequently classified as having “some concerns.”

The measurement of outcomes (Domain 4) was consistently rated as low risk across studies. This was primarily due to the widespread use of objective and standardized measures, including instrumented gait assessments, neurophysiological recordings and computerized behavioral tasks (e.g., Broeder et al., 2023; Conceição et al., 2021). These approaches reduce the likelihood of measurement bias.

The domain of selection of the reported result (Domain 5) was identified as the most problematic. A considerable number of studies did not report trial preregistration or provided insufficient information to verify whether outcomes were prespecified (e.g., Doruk et al., 2014; Manenti et al., 2016). This limitation resulted in a frequent classification of “some concerns.” In contrast, studies that included prospective registration (e.g., Lawrence et al., 2018; Wong et al., 2024) were rated as low risk in this domain.

Overall, these findings indicate a moderate level of methodological quality, with clear improvements observed in more recent studies but persistent limitations in reporting transparency and methodological detail.

## 4 Discussion

This systematic review provides a comprehensive synthesis of the effects of transcranial direct current stimulation (tDCS) on memory and learning processes in Parkinson’s disease. Overall, the evidence suggests potential benefits of anodal stimulation, particularly over the dorsolateral prefrontal cortex (DLPFC) in multi-session protocols combined with cognitive or motor training. However, these effects are heterogeneous and not consistently observed across studies, especially in single-session paradigms. Importantly, the methodological quality of the included studies, as indicated by the ROB-2 assessment, suggests that the overall certainty of the evidence is moderate and warrants cautious interpretation.

### 4.1 Interpretation of the findings

Interpretation of these results should be situated within the pathophysiological framework of PD, characterized by dysfunction of fronto-striatal circuits and alterations in long-term potentiation and depression (LTP/LTD) mechanisms (Obeso et al., 2008; Lovinger, 2010; Madadi Asl et al., 2022). Anodal stimulation of the DLPFC may support executive processes involved in working memory, facilitating the encoding and manipulation of relevant information (Lu et al., 2021; Robbins and Cools, 2014; Wischnewski et al., 2024). In the motor domain, stimulation of M1 during motor practice optimizes the synaptic reorganization involved in the acquisition and consolidation of procedural skills, which is consistent with previous studies on use-dependent plasticity (Barbati et al., 2020; Jin and Costa, 2015; Keitel et al., 2018). This interpretation is consistent with contemporary models of use-dependent plasticity, according to which stimulation most effectively modulates the circuits activated during task performance.

The results of this review are consistent with research documenting positive effects of tDCS on cognitive domains in neuropsychiatric populations and older adults (Cheng et al., 2020; Meléndez et al., 2023; Schwippel et al., 2025). In PD, studies such as Doruk et al. (2014) and Bueno et al. (2019) reported improvements in TMT-B performance and verbal fluency following DLPFC stimulation, which supports the hypothesis that prefrontal stimulation may enhance executive processes relevant to memory functioning in individuals with PD. Taken together, the available evidence suggests that anodal stimulation may increase the efficiency of task-relevant neural networks during performance.

These results can be contextualized against prior reviews addressing tDCS and cognition in PD. Suarez-García et al. (2020) found that, across the studies they meta-analyzed, the clearest signal was for executive function, whereas memory showed no measurable overall benefit. Consistent with this pattern, Cammisuli et al. (2022) concluded that tDCS may improve some executive functions in Parkinson’s disease, but that effects in other cognitive domains remain uncertain. Vandendoorent et al. (2023) similarly reported no statistically significant additional effect of tDCS over placebo on motor sequence learning, although they noted a trend suggesting that working memory training may benefit under some protocols. The present review extends these prior syntheses by focusing specifically on memory- and learning-related outcomes, and its findings similarly point to domain- and protocol-dependent effects rather than a uniform benefit of tDCS.

Studies integrating tDCS with physical exercise or dual-task conditions (Conceição et al., 2021; Wong et al., 2024) also reported benefits in processing speed and functional performance, suggesting that the combination of stimulation and activity may enhance efficacy possibly through mechanisms related to arousal and cortical activation that are fundamental to memory- and learning-related outcomes. These findings are consistent with the possibility that tDCS may yield greater benefits when administered in conjunction with activities involving memory and attention processes, reinforcing the idea of a synergistic effect.

In the motor domain, Broeder et al. (2019) 2023 demonstrated that tDCS over M1, combined with writing training, improved retention and transfer in learning processes, particularly when combined with dopaminergic medication, suggesting that pharmacological interactions may be relevant to plasticity. However, discrepancies remain: Simpson and Mak (2022) found no effects on explicit motor learning with anodal polarity, whereas cathodal or placebo conditions showed improvements, raising questions about polarity dependence, the functional state of the network, and individual factors such as the degree of cognitive impairment or the ON/OFF medication state.

Therefore, although converging patterns of positive findings were identified, the evidence was not entirely consistent. Trials combining tDCS with cognitive or physical training reported improvements in processing speed, verbal fluency, and functional performance. However, some studies found no significant effects or reported polarity-dependent differences, indicating that the response to tDCS may be modulated by individual variables such as dopaminergic medication state (ON/OFF), degree of cognitive impairment, or the functional integrity of the targeted circuits. These discrepancies likely reflect methodological differences across studies, including variation in stimulation parameters and the characteristics of the tasks used to induce learning.

### 4.2 Factors modulating treatment response

The results of this review highlight several factors that may modulate the effects of tDCS in PD. The number of sessions appears to be a critical factor. Multi-session protocols and combination with training seem to be more effective than isolated sessions. Furthermore, it should be considered that the cortical target must be tailored to the objective (DLPFC for declarative memory and executive functions or M1 for procedural learning and motor tasks). Methodological variability in polarity, intensity, and montage may account for some of the discrepancies across studies, underscoring the need for standardized protocols and personalization based on neurocognitive profiles.

These findings are relevant for neurorehabilitation programs, as they suggest the potential utility of tDCS in the design of integrated interventions combining stimulation with cognitive and/or physical training. They also open the possibility of future implementation in home-based protocols, may support scalability in clinical settings, and could improve rehabilitation outcomes in clinical practice.

Overall, tDCS emerges as a promising tool for enhancing memory and learning processes in PD, particularly in multi-session protocols and when combined with training. However, methodological heterogeneity and the limitations in the current evidence preclude firm conclusions and underscore the need for rigorous research to establish clinically meaningful recommendations based on robust evidence.

### 4.3 Limitations and future research

This review provides an overview of the current state of evidence regarding tDCS in cognitive and motor domains associated with learning and memory in Parkinson’s disease. Several limitations should be acknowledged. Methodological heterogeneity across studies makes direct comparison difficult and limits the ability to derive recommendations for clinical application. In addition, some studies have small sample sizes.

The ROB-2 assessment suggests that, although the methodological quality of studies investigating transcranial direct current stimulation (tDCS) in Parkinson’s disease has improved over time, several structural limitations remain and should be carefully considered when interpreting the evidence.

The most prominent limitation across studies was related to the selection of reported results, primarily due to the lack of preregistration or insufficient reporting of predefined outcomes (Doruk et al., 2014; Manenti et al., 2016). This issue raises concerns regarding selective reporting and analytic flexibility, particularly in studies with multiple behavioral and neurophysiological outcomes.

Another important limitation concerns the incomplete reporting of randomization procedures, especially in crossover designs (Swank et al., 2016; Lau et al., 2019). Although crossover studies are common in tDCS research due to their efficiency, insufficient methodological description limits confidence in allocation procedures and may introduce bias.

Blinding was generally well implemented using standardized sham protocols; however, several studies relied on single-blind designs or did not explicitly ensure evaluator blinding (Firouzi et al., 2021; Simpson and Mak, 2022). This is particularly relevant in behavioral outcomes, where expectancy effects may influence performance.

Despite these limitations, the body of evidence presents notable strengths. Most studies employed objective and instrumented outcome measures, such as gait analysis systems (e.g., GAITRite), reaction time paradigms, or neurophysiological techniques, which minimize measurement bias (Conceição et al., 2021; Broeder et al., 2023). Additionally, attrition rates were generally low, supporting the reliability of outcome data.

Importantly, a clear trend toward improved methodological rigor is observed in recent trials (2021–2024), which increasingly incorporate randomized controlled designs, double blinding, and trial preregistration (Mishra and Thrasher, 2022; Wong et al., 2024). These improvements suggest a positive evolution in the field. Nevertheless, persistent issues such as small sample sizes, incomplete reporting, and variability in study design indicate that current findings should be interpreted with caution.

Taken together, while the existing literature provides valuable insights into the potential effects of tDCS, future studies should prioritize preregistration, transparent reporting, and adequately powered randomized controlled designs to strengthen the overall evidence base.

Another important limitation concerns the considerable heterogeneity in stimulation protocols across studies, including differences in electrode montage, stimulation intensity, polarity, session duration, number of sessions, and cortical targets. This variability complicates the identification of optimal therapeutic parameters and limits reproducibility across studies.

Furthermore, many studies did not adequately control for potentially confounding variables such as dopaminergic medication status (ON/OFF phases), disease severity, cognitive baseline, fatigue, sleep quality, or psychiatric symptoms such as depression and apathy, all of which may influence cognitive performance and responsiveness to tDCS.

A further limitation is the inconsistency in outcome measures. Although several studies assessed executive functions, working memory, or procedural learning, the neuropsychological instruments varied substantially, making quantitative comparisons difficult and preventing the establishment of standardized cognitive outcomes for PD-related learning and memory.

Another relevant issue is the predominance of short-term assessments. Most studies evaluated outcomes immediately after stimulation or within a few weeks, providing limited information regarding the durability and long-term clinical relevance of the observed effects. Consequently, it remains unclear whether improvements translate into sustained functional benefits in daily life.

Additionally, few studies incorporated neurophysiological or neuroimaging measures capable of elucidating the neural mechanisms underlying cognitive improvement. The absence of biomarkers limits understanding of how tDCS modulates cortical and subcortical plasticity in Parkinson’s disease.

The review is also limited by the possibility of publication bias, as studies reporting positive findings are more likely to be published than those reporting null or negative results. This may contribute to an overestimation of the efficacy of tDCS.

Another limitation relates to sample representativeness. Most participants presented mild-to-moderate PD and relatively preserved cognition, which restricts the generalizability of findings to patients with advanced disease stages or Parkinson’s disease dementia.

Finally, language restrictions to English and Spanish publications may have excluded relevant studies published in other languages, potentially limiting the comprehensiveness of the review.

Future studies should prioritize the development of standardized stimulation protocols to facilitate replication and comparison between trials. Establishing consensus regarding optimal intensity, polarity, electrode placement, session frequency, and treatment duration will be essential for clinical translation.

Large-scale randomized controlled trials with adequate statistical power are needed to confirm the efficacy of tDCS on memory and learning in PD and to identify which patient subgroups benefit most from stimulation.

Future research should also investigate individualized approaches to tDCS based on patient characteristics such as disease stage, cognitive profile, medication response, and neurophysiological markers. Personalized neuromodulation may optimize treatment efficacy and reduce variability in outcomes.

Longitudinal studies with extended follow-up periods are necessary to determine the persistence of cognitive and motor improvements and to assess whether repeated stimulation can slow cognitive decline or improve functional independence over time.

Future randomized controlled trials should incorporate repeated assessments at baseline, immediately post-intervention, and during medium- and long-term follow-up periods (e.g., 1, 3, and 12 months) to determine the durability and clinical relevance of cognitive and motor benefits.

Another important direction involves combining tDCS with multimodal interventions such as cognitive rehabilitation, physical exercise, gait training, virtual reality, or computerized cognitive training. Exploring synergistic effects may enhance neuroplasticity and improve ecological validity.

Future investigations should incorporate neuroimaging and electrophysiological techniques such as functional MRI, EEG, or TMS-EEG to better understand the neural mechanisms associated with tDCS-induced cognitive changes and identify biomarkers predictive of treatment response.

Research should also examine the influence of dopaminergic medication states and neurotransmitter interactions on stimulation efficacy, as these factors may critically modulate cortical plasticity and behavioral outcomes.

Moreover, greater attention should be paid to ecological and functional outcomes, including activities of daily living, quality of life, autonomy, and caregiver burden, rather than relying exclusively on laboratory-based cognitive measures.

Another avenue worthy of further investigation is the exploration of home-based and remotely supervised tDCS protocols, which may improve accessibility, adherence, and scalability in clinical populations with mobility limitations.

Finally, future systematic reviews and meta-analyses should conduct quantitative analyses stratified by stimulation parameters, cognitive domains, and disease characteristics to better clarify moderators of treatment efficacy and establish evidence-based clinical guidelines.

### 4.4 Clinical implications

tDCS may represent a potentially effective complementary approach for improving learning and memory processes in individuals with Parkinson’s disease; it may be particularly useful when combined with specific training programs.

The current proliferation of home-use devices could facilitate its application, particularly in individuals with Parkinson’s disease. However, well-defined and supervised application protocols would be essential.

## 5 Conclusion

This systematic review synthesized current evidence regarding the effects of transcranial direct current stimulation (tDCS) on learning- and memory-related processes in individuals with Parkinson’s disease. Overall, the findings suggest that tDCS is a potentially beneficial noninvasive neuromodulatory intervention with the potential to improve cognitive and motor domains associated with neuroplasticity, particularly working memory, verbal fluency, executive functioning, procedural learning, and motor learning consolidation.

The evidence indicates that the most consistent benefits are observed when anodal stimulation is applied over the dorsolateral prefrontal cortex (DLPFC) or primary motor cortex (M1) using multi-session protocols combined with cognitive or motor training. In contrast, single-session interventions generally produced limited or inconsistent effects, reinforcing the importance of repeated stimulation and task-dependent plasticity mechanisms. The combination of tDCS with cognitive rehabilitation, dual-task training, or physical exercise appears to enhance therapeutic effects, suggesting a synergistic interaction between stimulation and behavioral activation.

Despite these promising findings, the current evidence remains limited by methodological heterogeneity, small sample sizes, variability in stimulation parameters, inconsistent outcome measures, and the scarcity of long-term follow-up studies. These limitations preclude definitive conclusions regarding the optimal stimulation protocols and the generalizability of the results to broader Parkinson’s disease populations.

Nevertheless, the reviewed studies support the potential role of tDCS as an adjunctive therapeutic tool within neurorehabilitation programs aimed at improving learning and memory processes in Parkinson’s disease. Future large-scale, methodologically rigorous, and longitudinal studies are needed to establish standardized protocols, clarify the neurobiological mechanisms underlying treatment effects, and determine the long-term clinical relevance of tDCS in cognitive and motor rehabilitation for individuals with Parkinson’s disease.

## Data Availability

The original contributions presented in the study are included in the article/supplementary material, further inquiries can be directed to the corresponding author.

## Footnotes

1
https://www.rayyan.ai/
